# Circulating cell free DNA as the diagnostic marker for colorectal cancer: a systematic review and meta-analysis

**DOI:** 10.18632/oncotarget.25314

**Published:** 2018-05-11

**Authors:** Xin Wang, Xia-Qing Shi, Peng-Wei Zeng, Fong-Ming Mo, Zi-Hua Chen

**Affiliations:** ^1^ Department of General Surgery, Xiangya Hospital of Central South University, Changsha, China; ^2^ Key Laboratory of Nanobiological Technology of Chinese Ministry of Health, Xiangya Hospital of Central South University, Changsha, China; ^3^ Department of General Surgery, Xiangya Hospital of Central South University, Changsha, China; ^4^ Key Laboratory of Nanobiological Technology of Chinese Ministry of Health, Xiangya Hospital of Central South University, Changsha, China; ^5^ Department of General Surgery, Xiangya Hospital of Central South University, Changsha, China

**Keywords:** circulating cell free DNA, colorectal cancer, diagnosis, meta-analysis

## Abstract

**Background:**

Quantitative analyses of circulating cell-free DNA (cfDNA) are suggested to be a promising method for the detection of colorectal cancer, validated clinical relevance of cfDNA has not been published so far. Though some of the inconsistent results were published. This study is the first meta-analysis to systematically evaluate the diagnostic accuracy of circulating cfDNA as non-invasive biomarkers for colorectal cancer.

**Results:**

Fourteen studies concerning a quantitative analysis of circulating cfDNA for the diagnosis of colorectal cancer met the inclusion criteria. Data includes 1,258 patients with colorectal cancer and 803 healthy individuals as control was analyzed. The summary estimates were as follow: sensitivity, 0.735 (95% CI 0.713–0.757); specificity, 0.918 (95% CI, 0.900–0.934); positive likelihood ratio, 8.295 (95% CI, 5.037–13.659); negative likelihood ratio, 0.300 (95% CI, 0.231–0.391); diagnostic odds ratio, 30.783 (95% CI, 16.965–55.856); and area under the curve, 0.8818 (95% CI, 0.88–0.93), respectively. Publication bias was not evident with Deeks’ funnel plot asymmetry test (*p* = 0.197).

**Materials and Methods:**

A systematic literature was searched in PubMed, EMBASE, Cochrane Library and Chinese National Knowledge Infrastructure from their inception to August 07, 2017. Analyses were conducted by Meta-DiSc 1.4 and Stata 12.0. Diagnostic accuracy in sensitivity, specificity and aspects were pooled. Subgroup analyses and meta-regression were performed to identify the sources of heterogeneity. Clinical utility of the cfDNA was evaluated by Fagan nomogram.

**Conclusions:**

Our meta-analysis suggested that the diagnostic accuracy of circulating cfDNA has unsatisfactory sensitivity but acceptable specificity for diagnosis of colorectal cancer. Furthermore, the integrity index (ALU247/ALU115) is better than absolute DNA concentration in diagnostic accuracy of colorectal cancer.

## INTRODUCTION

Colorectal cancer is the third most common cancer worldwide, with 945,000 new cases diagnosed and 492,000 deaths each year. This cancer has vague or nonspecific symptoms, so it is generally diagnosed in the advanced stage. Furthermore, mortality of colorectal cancer is strongly related to disease stage: the 5-year survival rate decreases from 95% in stage I to 6% in patients with stage IV [1]. Therefore, methods to improve early detection of colorectal cancer in specificity and sensitivity are a critical need.

Currently, the major available strategies for diagnosis of colorectal cancer include colonoscopy and fecal occult blood testing. Histopathology examination via colonoscopy is considered as the golden standard. However, people always reject screening of colonoscopy because of its uncomfortable invasive process and complex bowel preparation in China. In addition, fecal occult blood testing, even colonoscopy, may fail to detect carcinomas at early stage. Blood-based tests are the most promising method, as getting a patient's blood is a easy and convenient way of early examination.

Carcinoembryonic antigen (CEA) and carbohydrate antigen-19-9 (CA 19-9) etc are clinically used as routine tumor markers to monitor disease progression of colorectal cancer. Nevertheless, these markers have limited use in early diagnosis and cancer screening due to their low sensitivity and specificity [2]. Abnormal results of the above tumor markers have been shown in cancer-free patients who suffer from other diseases Thus, searching for new blood biomarkers to diagnose colorectal cancer is attracted many researchers.

Circulating Cell Free DNA (cfDNA) is a type of cell-free nucleic acids that is released from normal and deceased cells from apoptosing and necrotizing processes [3]. Moreover, the expression of cfDNA is usually altered in malignancies, even in early phase. Recently, some studies reported that quantitative analysis of circulating cfDNA has led an interest as a potential biomarkers for clinical applications and played an important role in assessing tumor progression and predicting prognosis [4], diagnosis and response to treatment in several types of cancers including colorectal cancer. CfDNA can be detected in the peripheral blood, but the origins of cfDNA are controversial. Studies have suggested that the level of cfDNA is increased in both cancer patients and in various non-malignant pathological conditions compared to healthy individuals [5]. The cfDNA fragments released from necrotic tumor cells differs in size, whereas cfDNA released from apoptotic non-tumor cells are consistent and truncated measuring 185-200 base pairs in length [6]. Therefore, most studies used ALU115 and ALU247 fragments for cfDNA measurement, ALU 115 represent total DNA (longer and shorter fragments of cfDNA) and ALU247 represent tumor DNA (longer fragments of cfDNA). More specific approaches have been proposed, such as integrity index, which describes the relation between longer and shorter DNA fragments is obtained by calculating the ratio of ALU247 to ALU115 [7].

Validated clinical relevance of cfDNA has not been published so far. Though some of the inconsistent results were published. The present study aimed to carry out the first meta-analysis to quantitatively analyze the diagnostic accuracy of circulating cfDNA and to systematically evaluate the potential of circulating cfDNA as non-invasive biomarkers for colorectal cancer. We also sought to compare the integrity index and the concentration of cfDNA in the diagnosis of colorectal cancer.

## RESULTS

### Characteristics of included studies and diagnostic accuracy

**Figure 1:**
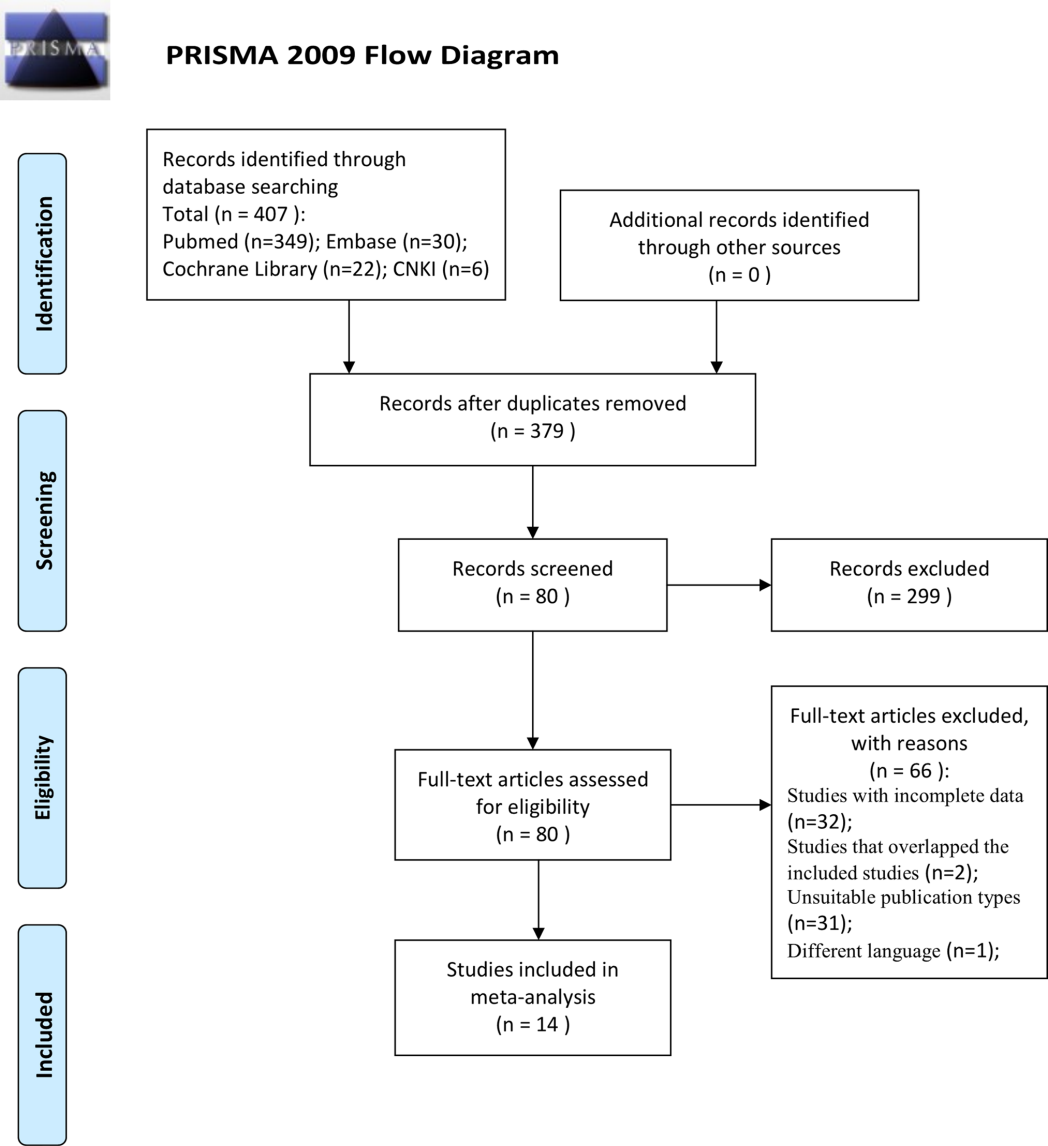
Flowchart showing selection of studies for inclusion in the meta-analysis

The process used to select studies is summarized in Figure 1. In this study, we only focus on the cfDNA from blood sample without mutant and methylation gene as biomarkers. Fourteen studies [7–20] concerning a quantitative analysis of circulating cfDNA for the diagnosis of colorectal cancer that met the inclusion criteria were identified from 407 publications, including a total of 1,258 patients with colorectal cancer and 803 healthy control individuals. All the colorectal cancer patients were diagnosed based on histopathological examination. The general characteristics of these studies are shown in Table 1.

**Table 1:** Characteristics of 14 studies for quantitative analysis of circulating cell free DNA in the diagnosis of colorectal cancer

Study	Country	Year	No. of P/C	Assay method	Tumor stage	Sample	Measuring object	Cut off	TP	FP	FN	TN	Sensitivity (%)	Specificity (%)
**Flamini, E.**	Italy	2006	75/75	qPCR	Dukes A-D	serum	CFD levels	12.5 ng/Ml	61	20	14	55	81.3%	73.3%
**Umetani, N.**	USA	2006	32/51	qPCR	I-IV	serum	CFD levels	stage I/II 1.63 III/IV 1.73 ng/uL	13	5	19	46	41	90
							ALU 247/115	0.22 ng/uL	18	5	14	46	56	90
**Pucciarelli, S.**	Italy	2009	136/55	qPCR	I-IV	plasma	ALU115	4.86 ng/ml	107	8	29	47	78.52	86.08
							ALU247	3.04ng/ml	106	10	30	45	77.94	82.28
**Danese, E.**	Italy	2010	118/26	RT-PCR	I-IV	Serum	CFD levels	37 ng/mL	98	2	20	24	82.9	92.3
**Agostini, M.**	Italy	2011	67/35	qPCR	0-IV	Plasma	Alu 247	2.0 ng/ml	63	0	4	35	94	100
**Czeiger, D.**	Israel	2011	38/34	SYBR Gold Nucleic Acid Gel Stain	I-IV	serum	CFD levels	841 ng/ml	16	2	22	32	42	94
**Zhang.GH**	China	2012	61/92	bDNA	I-IV	serum	CFD levels	768.9ng/ml	43	1	18	91	70.5	98.9
**Jiang.WQ**	China	2012	178/56	RT-PCR	I-III	plasma	CFD levels	32.78 ng/ml	86	3	92	53	48.1	94.3
**Qi, J.**	China	2013	31/92	bDNA	I-IV	serum	Alu-based	634.9 ng/ml	20	1	11	91	64.5	98.9
**Leszinski, G.**	Germany	2014	24/24	RT-PCR	Not given	serum	Alu-based	ALU115: 1.31	18	7	6	17	75	70.8
								ALU247: 1.29						
**Hao, T. B.**	China	2014	104/110	qPCR	I-IV	Serum	ALU115	694.0 ng/ml	72	1	32	109	69.23	99.09
							ALU247/115	0.52	76	3	28	107	73.08	97.27
**El-Gayar, D.**	Egypt	2016	50/20	RT-PCR	II-IV	Serum	CFD levels	3.3ng/μl	34	7	16	13	68	65
							ALU 247/115	0.41	45	3	5	17	90	85
**Berger, A. W.**	Germany	2017	15/38	Qubit dsDNA HS Assay Kit	IV	plasma	CFD levels	7.21 ng/ml	14	3	1	35	93.3	92.1
**Lan, Y. T.**	China	2017	329/95	qPCR	I-IV	Serum	CFD levels	2,700 copies/ml	272	4	57	91	82.7	95.8

Abbreviations: No. of P/C = number of patients/control; RT/q-PCR = real-time/quantitative PCR; bDNA = branched DNA; TP = true positive; FP = false positive; FN = false negative; TN = true negative. CFD = cell-free DNA.

Based on the QUADAS-2, the quality assessment results of the eligible fourteen studies are shown in Table 2. To some extent, the overall quality of these included studies were generally robust.

**Table 2:** The Quality Assessment of Diagnostic Accuracy Studies-2 (QUADAS-2)

Study	Risk of Bias				Applicability Concerns		
	Patient Selection	Index Test	Reference Standard	Flow and Timing	Patient Selection	Index Test	Reference Standard
Flamini, E. 2006	H	U	U	U	L	L	U
Umetani, N. 2006	L	U	L	H	L	L	L
Pucciarelli, S. 2009	U	U	U	U	U	U	U
Danese, E. 2010	U	U	L	U	L	L	L
Agostini, M. 2011	H	L	L	H	U	L	L
Czeiger, D. 2011	L	L	U	H	L	L	L
Zhang, GH. 2012	U	U	U	U	L	U	L
Jiang, WQ. 2012	H	U	L	U	U	L	L
Qi, J. 2013	L	U	L	L	L	L	L
Leszinski, G. 2014	U	U	U	H	U	U	U
Hao, T. B. 2014	L	U	L	L	L	L	L
El-Gayar, D. 2016	L	L	L	L	L	L	L
Berger, A. W. 2017	L	L	U	L	L	L	L
Lan, Y. T. 2017	L	L	U	L	L	L	U

Abbreviations: L, low risk; H, high risk; U, unclear risk.

**Figure 2:**
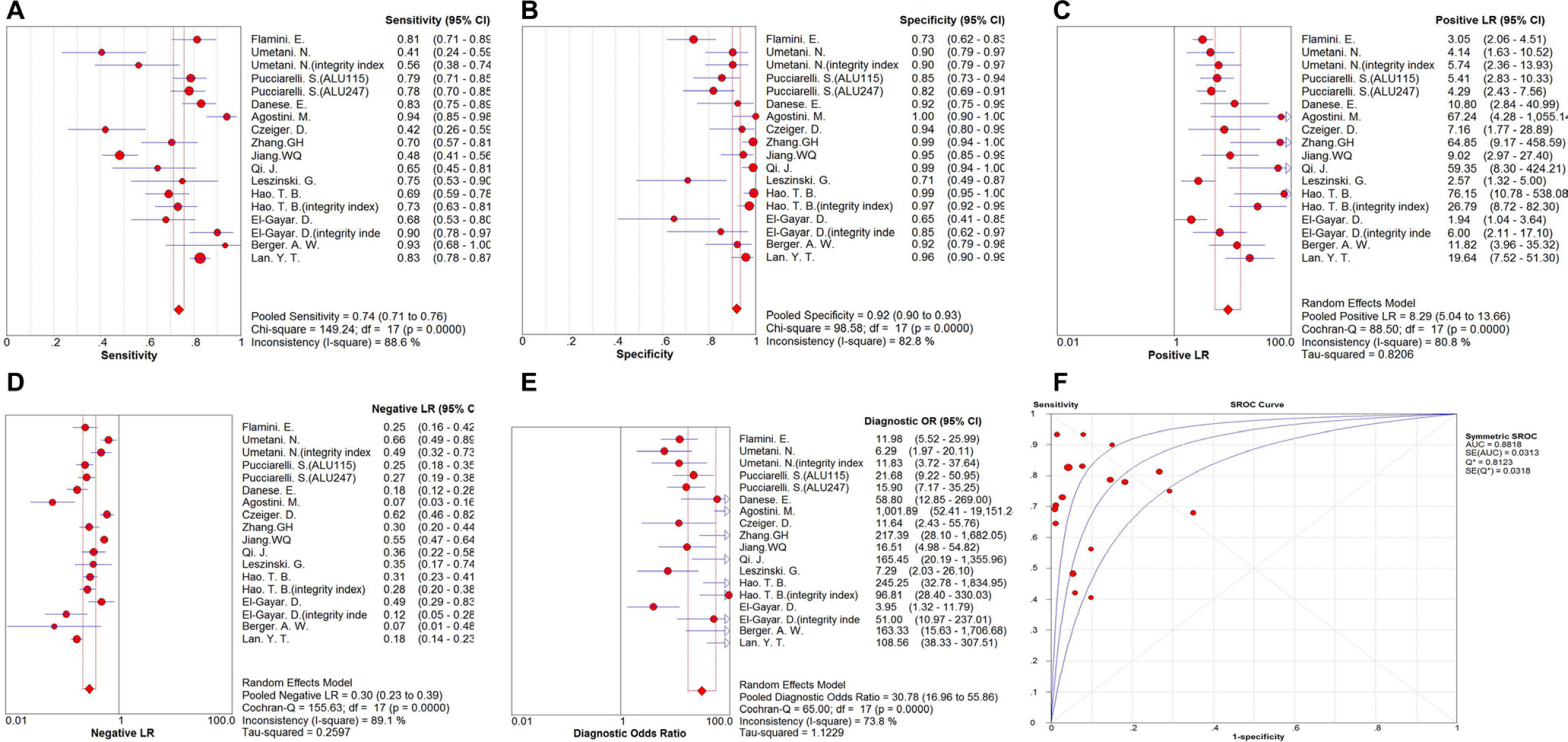
Forest plot of the overall pooled (**A**) sensitivity; (**B**) specificity; (**C**) PLR;(**D**) NLR; (**E**) DOR for quantitative analysis of circulating cell free DNA in the diagnosis of colorectal cancer (**F**). The SROC curve for quantitative analysis of circulating cell free DNA in the diagnosis of colorectal cancer.

Eighteen sets of data were included in the analysis, significant heterogeneity existed among the overall pooled results (I_2_ for sensitivity was 88.6%, *p* = 0.000 and I_2_ for specificity was 82.8%, *p* = 0.000). The threshold effect was the major cause of heterogeneity. When it existed, the logit of sensitivity were positively correlated with the logit of 1-specificity, and there would be shoulder-like ROC plane curve. In this meta-analyses, the Spearman correction coefficient was 0.096 and the *p* value was 0.705, confirming that the threshold effect was not significant and the heterogeneity must be caused by other reasons. Therefore, we could combine most evaluation index directly. The overall pooled sensitivity and specificity were 0.735 (95% CI 0.713–0.757) and 0.918 (95% CI, 0.900–0.934), respectively. Forest plots are shown in Figure 2. In addition, the overall pooled PLR was 8.295 (95% CI, 5.037–13.659), NLR was 0.300 (95% CI, 0.231–0.391) and DOR was 30.783 (95% CI, 16.965–55.856) (Figure 2). Cochran-Q = 65.00, *p* = 0.0000 and the distribution of DORs does not along a straight line, which means heterogeneity exist due to non-threshold effect. The SROC curve for the included studies is shown in Figure 2. The AUC was 0.8818 (95% CI, 0.88–0.93), indicating a relatively high diagnostic accuracy of circulating cfDNA for colorectal cancer.

Subgroup analyses of studies included measuring objects (integrity index:ALU247/ALU115 or ALU115&cfDNA levels), participants (China, Italy or other countries), specimen types (plasma or serum) and sample size (number of cases ≥ 100 or number of cases < 100). We found that integrity index: ALU247/ALU115 group had a better diagnostic accuracy compared with ALU115&cfDNA levels group, even overall data, with sensitivity of 0.747 versus 0.717 (ALU115&cfDNA levels) and 0.735 (overall), specificity of 0.939 versus 0.917 (ALU115&cfDNA levels) and 0.918 (overall), PLR of 9.398 versus 8.235 (ALU115&cfDNA levels) and 8.295 (overall), NLR of 0.277 versus 0.334 (ALU115&cfDNA levels) and 0.300 (overall), DOR of 37.767 versus 27.825 (ALU115&cfDNA levels) and 30.783 (overall) and AUC of 0.9275 versus 0.8652 (ALU115&cfDNA levels) and 0.8818 (overall), respectively. We also found that China has the best overall accuracy in detecting colorectal cancer than Italy or other country group by current evidence. with sensitivity (China 0.705, Italy 0.818, other country 0.656), specificity (China 0.977, Italy 0.837, other country 0.866), PLR (China 24.618, Italy 5.200, other country 4.269), NLR (China 0.312, Italy 0.212, other country 0.416), DOR (China 89.386, Italy 25.453, other country 12.084) and AUC (China 0.9293, Italy 0.8688, other country 0.8667). Furthermore, We cannot determine which is more accurate in serum-based assays or plasma -based assays, sensitivity of 0.750 versus 0.707, specificity of 0.924 versus 0.900, PLR of 8.858 versus 6.868, NLR of 0.324 versus 0.214, DOR of 29.789 versus 31.501 and AUC of 0.8581 versus 0.9365. In addition, the subgroup with larger sample size own a higher potential diagnostic value of cfDNA than smaller sample size group, with sensitivity (0.739 versus 0.726), specificity (0.939 versus 0.898), PLR (11.397 versus 6.390), NLR (0.273 versus 0.319), DOR (43.554 versus 23.910) and AUC (0.8932 versus 0.8772). The pooled data such as sensitivity, specificity, PLR, NLR, DOR, and AUC for each subgroup are shown in Table 3A. I_2_ and *p* values for individual subgroup analysis are shown in Supplementary Table 1.

**Table 3A:** Results of the subgroup analyses performed to identify potential sources of heterogeneity

Variables	No. of data	Sensitivity	Specificity	PLR	NLR	DOR	AUC
**Overall**	18	0.735 (0.713-0.757)	0.918 (0.900-0.934)	8.295 (5.037-13.659)	0.300 (0.231-0.391)	30.783 (16.965-55.856)	0.8818
**Measuring object ALU247/ALU115**	3	0.747 (0.679-0.808)	0.939 (0.894-0.969)	9.398 (3.413-25.875)	0.277 (0.149-0.516)	37.767 (9.912-143.90)	0.9275
**ALU115&CFDlevels**	13	0.717 (0.691-0.742)	0.917 (0.895-0.935)	8.235 (4.399-15.414)	0.334 (0.246-0.454)	27.825 (13.371-57.903)	0.8652
**Country China**	6	0.705 (0.672-0.736)	0.977 (0.960-0.987)	24.618 (13.119-46.195)	0.312 (0.202-0.480)	89.386 (38.281-208.71)	0.9293
**Italy**	5	0.818 (0.782-0.850)	0.837 (0.785-0.881)	5.200 (2.828-9.564)	0.212 (0.155-0.290)	25.453 (11.234-57.669)	0.8688
**Other countries**	7	0.656 (0.592-0.715)	0.866 (0.816-0.906)	4.269 (2.603-6.999)	0.416 (0.280-0.618)	12.084 (5.547-26.324)	0.8667
**Sample Plasma**	5	0.707 (0.666-0.745)	0.900 (0.854-0.935)	6.868 (3.927-12.013)	0.214 (0.099-0.460)	31.501 (12.377-80.176)	0.9365
**serum**	13	0.750 (0.723-0.776)	0.924 (0.903-0.941)	8.858 (4.435-17.691)	0.324 (0.244-0.431)	29.789 (13.668-64.923)	0.8581
**sample size ≥ 100**	7	0.739 (0.712-0.765)	0.939 (0.914-0.958)	11.397 (5.602-23.186)	0.273 (0.184-0.405)	43.554 (20.432-92.843)	0.8932
**< 100**	11	0.726 (0.684-0.766)	0.898 (0.870-0.923)	6.390 (3.480-11.731)	0.319 (0.218-0.467)	23.910 (10.374-55.107)	0.8772

### Meta-regression analysis for heterogeneity

We performed a meta-regression analysis to explore possible sources of the heterogeneity from the articles.

We managed to separately evaluate the following specific variables for their effects on heterogeneity: “Publication year” (Year: before 2010 or after 2010), “Study location” (Country: China or Other countries), “type of specimens” (Sample: plasma or serum), “Methods of detection”(Assay methods: qPCR or non qPCR), measuring objects (Object: integrity index or others), number of cases (Size: ≥ 100 or < 100) and “four key domains in QUADAS-2”(Quality: with or without high risk of “Patient selection”, “Index Test”, “Golden Standard” and “Process and Progress”). Then, we carry out new regression analyses respectively after dropping the variables one by one, according to the *p* value from high to low. It was noticed that quality cause statistically significant differences among studies, indicating that quality substantially affect the diagnostic accuracy. The diagnostic accuracy of studies which are defined as high risk of bias is 0.25 times lower than studies had low and unclear risk of bias (RDOR = 0.25, 95% CI: 0.09–0.72; *p* = 0.0139). Other factors did not show any definite influence on heterogeneity (Table 3B).

**Table 3B:** Results of the meta-regression performed to identify potential sources of heterogeneity

Variance	Coefficient	Std. Err.	*p* - value	RDOR	[95% CI]
Sample	−0.613	0.8780	0.5029	0.54	(0.07; 3.95)
method	0.482	1.3205	0.7235	1.62	(0.08; 32.11)
object	−0.415	0.8626	0.6421	0.66	(0.09; 4.65)
quality	−1.054	0.8773	0.2601	0.35	(0.05; 2.54)
country	1.098	1.3648	0.4420	3.00	(0.14; 65.70)
size	−0.315	1.2322	0.8041	0.73	(0.04; 11.85)
year	0.161	0.8811	0.8594	1.17	(0.16; 8.62)
quality^*^	−1.376	0.4944	0.0139	0.25	(0.09; 0.72)

Abbreviations: Std.Er, standard error; CI, confidence interval. ^*^after processing.

### Clinical utility assessment

**Figure 3:**
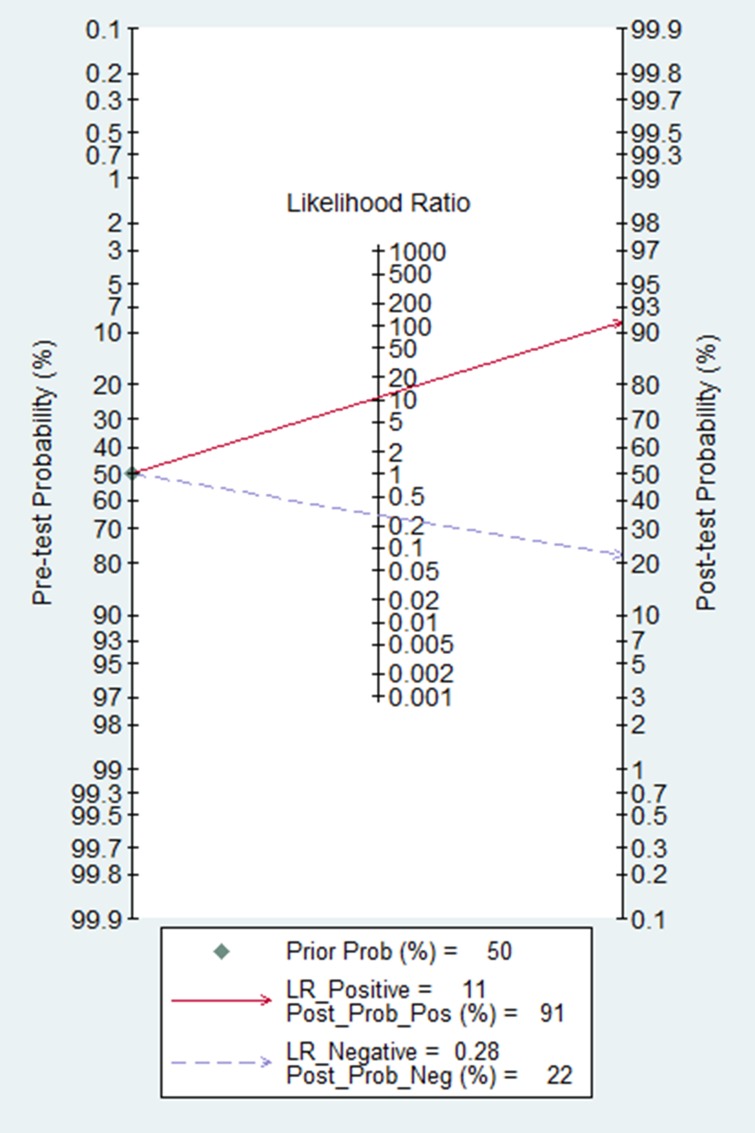
The Fagan nomogram for the assessment of clinical utility on circulating cell free DNA

The Fagan nomogram is a graphical tool for estimating how much the result on a diagnostic test changes the probability that a patient has a disease. To use this tool, you need to provide the probability of disease before testing and the likelihood ratio for the diagnostic test. From our Fagan's Nomogram (Figure 3), we found that when 50% was selected as the pre-test probability, in other word, the probability that a man suffer from the colorectal cancer was 50% via evaluation. After the calculation is done, the post-test probability would raise to 91% with a positive likelihood ratio of 11, and the probability would decrease to 22%, and the negative likelihood ratio was 0.28.

### Publication bias estimate

**Figure 4:**
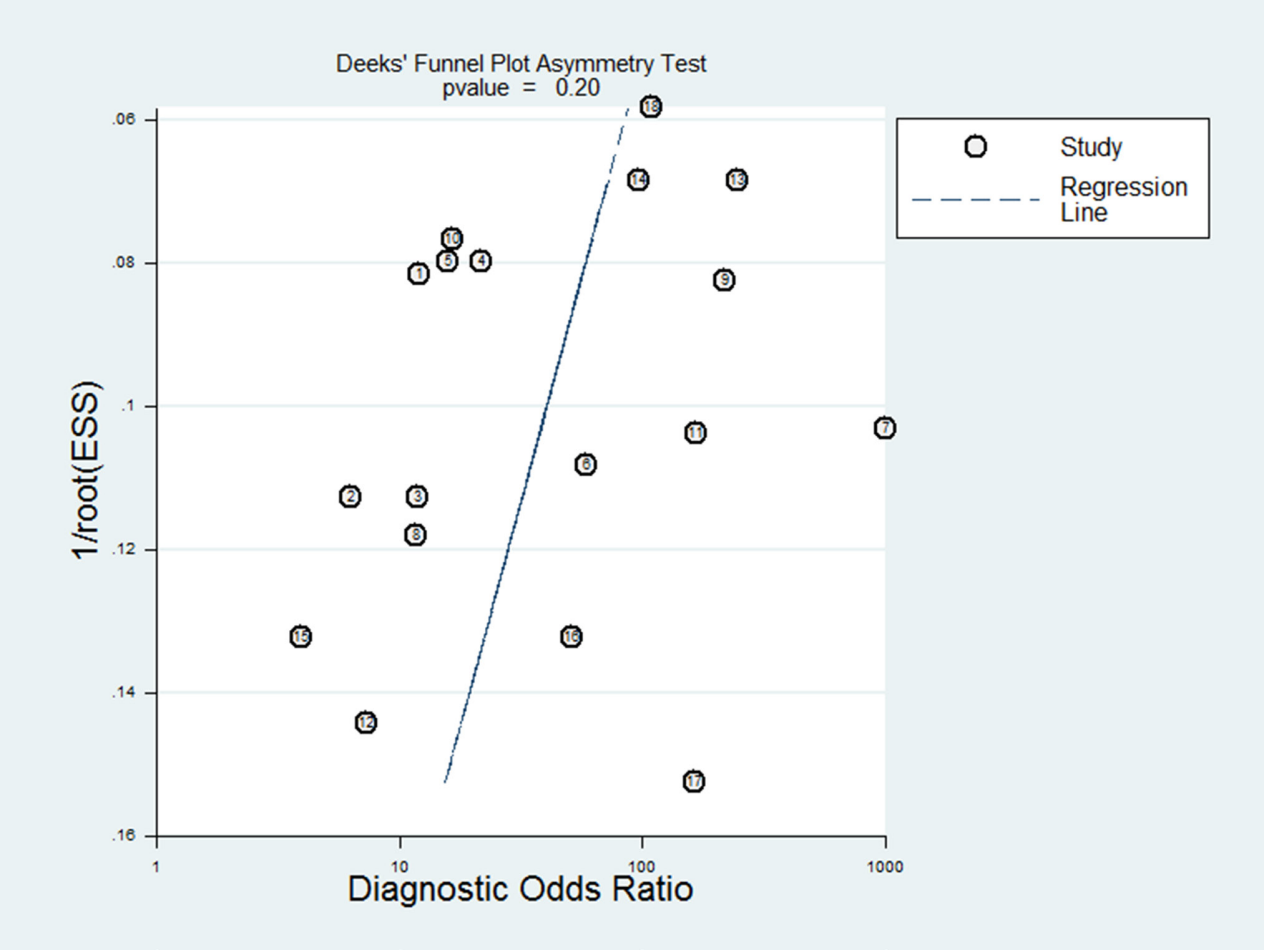
The Deeks’ funnel plot for the detection of publication bias of the included studies

Publication bias is evaluated visually by angle of regression line and horizontal axis (DOR axis) in the funnel plot. The angle should close to 90 degree when publication bias is absent. In this meta-analysis, publication bias was not evident with Deeks’ funnel plot asymmetry test (*p* = 0.197) (Figure 4).

## DISCUSSION

In 1948, Mandel and Metais firstly described the presence of cfDNA in human blood. Several years later, Leon, et al. [21] demonstrated cfDNA is associated with malignant tumors. Due to its higher level in cancer patients compared with healthy individuals, cfDNA has showed characteristics of a potential candidate biomarker of tumor response.

Later studies [22, 23] have reported that cfDNA in serum or plasma of cancer patient released from tumor necrotic are variable in length. In healthy individuals, the main source of cfDNA is apoptotic cells and DNA fragments is uniformly truncated into shorter fragments. Therefore, the amount of longer DNA fragments and the ratio between the longer and shorter fragments, known as the integrity index, may reflect the presence of cancer and become a promising alternative for early cancer screening, detection, and monitoring of treatment [3].

Several previous meta-analyses have published the diagnostic accuracy of quantitative analysis of cfDNA including ovarian cancer [24] and lung cancer [25]. Moreover, a meta-analysis [4] has revealed the significant prognostic values of cfDNA for RFS (HR: 2.78, 95% CI: 2.08–3.72) and OS (HR: 3.03, 95% CI: 2.51–3.66) in patients with colorectal cancer, but still lack systematically evaluation about colorectal cancer diagnosis. Hence, for the first time, we carried out this comprehensive meta-analysis to integrate all related publications and assess the accuracy of circulating cfDNA as a diagnostic biomarker for colorectal cancer.

In this exploratory meta-analysis of 14 studies, including 18 sets of data, the pooled sensitivity and specificity of the circulating cfDNA assay were 0.735 (95% CI 0.713–0.757) and 0.918 (95% CI, 0.900–0.934), respectively, indicating quantitative analysis of cfDNA has poor sensitivity but acceptable specificity for diagnosis of colorectal cancer. Likelihood ratios are used for assessing the value of performing a diagnostic test and the verity of sensitivity and specificity. LRs of greater than 10 may make a definite diagnosis for a disease, LRs of less than 0.1 may eliminate the possibility of a disease to some extent. LRs are more clinically meaningful than SROC curve and DOR. In our study, the pooled PLR and NLR of the circulating cfDNA assay was 8.295 (95% CI, 5.037–13.659) and 0.300 (95% CI, 0.231–0.391), respectively. This result suggested that colorectal cancer patients via circulating cfDNA assay have approximately 8.295 times higher chance have a positive result compared with healthy controls, and the probability of the individuals with colorectal cancer was approximately 30.0% when circulating cfDNA test was negative. These results indicated that the unsatisfactory likelihood ratios obtained in meta-analysis may reflect poor robustness and accuracy. DOR is commonly used to assess diagnostic efficiency because it combines sensitivity, specificity, PLR and NLR data. DOR indicates the multiples on the probability of a positive result versus a negative result in diagnostic test. The pooled DOR in our study was 30.783 (95% CI, 16.965–55.856), indicating a relatively high level of overall accuracy. Moreover, ROC is normally used to describe overall test performance and AUC serves as a measurement indicator, the AUC of SROC for cfDNA was 0.8818, indicating a relatively high accuracy of circulating cfDNA for colorectal cancer diagnosis.

We were able to separately evaluate four different subtypes. The studies on integrity index: ALU247/ALU115 group had a better overall accuracy compared with ALU115&cfDNA levels group, even overall data, with higher level of sensitivity, specificity, PLR, DOR and AUC. CfDNA reported by China has more accurate than cfDNA reported by Italy or other country group in diagnosis of colorectal cancer. Meanwhile, our subgroup analysis suggested that larger sample size groups were more accurate in detecting colorectal cancer than smaller sample size groups. We also found that serum-based assays showed a higher level of sensitivity, specificity and PLR but lower DOR and AUC compared with plasma-based assays. The Fagan nomogram reveal that incremental values of cfDNA could raise the probability of colorectal cancer from 50% to 91%, which means cfDNA is excellent in the clinical utility assessment.

Heterogeneity is an critical issue in meta-analysis. In our study, Significant heterogeneity was detected among the trials by the I^2^ test. The threshold effect is usually a primary cause of heterogeneity in diagnostic meta-analysis. However, the spearman correction coefficient of our study (0.096, *p* = 0.705 > 0.05) indicated that the heterogeneity must be caused by other reasons rather than threshold effect. In order to explore the potential source of heterogeneity, we investigated the characteristics of included studies such as publication year, study location, type of specimens, methods of detection, measuring objects, number of cases and four key domains in QUADAS-2 using meta-regression. Finally, our analysis revealed that study quality largely contributed to the substantial heterogeneity, indicating that the study design with high risk biases of “Patient selection”, “Index Test”, “Golden Standard” and “Process and Progress” could be easier than other characteristics to affect the diagnostic accuracy. Heterogeneity may also have risen due to other reasons, such as age, tumor type, metastasis, TNM staging, operation method and treatment protocol, which could not be analyzed in the present study due to the related data are so insufficient.

Although publication bias can be another problem in meta-analyses, Deeks’ funnel plot asymmetry test did not identify such bias, indicating that the results of our meta-analysis are reliable.

Many different hypotheses concerning the origin of the circulating cfDNA have been proposed, including liberation from the tumor itself by rupture or necrosis, a derivative from abnormal apoptotic pathways, autophagia, mitotic catastrophe and micrometastases [3].

However, injury, acute inflammation, or infarctions may also lead to cells rupture and cfDNA release [26]. In addition, fetal DNA can enter into the maternal bloodstream during pregnancy [27]. All these patterns may cause a false positive via increasing cfDNA level. There is no general agreement on the value of cfDNA measurement for patients with cancer. We still have no utter confidence in any subsequent recommendations on cfDNA. A future study will help determine this.

CEA and CA19-9 are widely used markers in clinical medicine for the diagnosis of CRC. In fact, increased CEA concentrations occur in only 5%–40% of CRC patients, and positive result are often observed in cancer-free patients who suffer from benign diseases such as liver damage or inflammatory diseases [10]; CA19-9 also have proven to be non-ideal [15]. Therefore, we hope and try to reveal that DNA integrity index or absolute DNA concentration could be a clinically useful surrogate markers. Because the related literatures and data are so insufficient that we had to give up analyzing the diagnostic value of cfDNA combine with the conventional tumor markers (CEA and CA19-9). We did not study whether combined CEA and circulating cfDNA could improves colorectal cancer screen.

Similar to all meta-analyses, our study was subject to several limitations. First, for the sources of substantial heterogeneity in our study, we could not identify its by subgroup analyses and meta-regression. Second, our study is limited because of the small sample size. Only 14 studies met our criteria to examine the quantitative analysis of circulating cfDNA for the diagnosis of colorectal cancer. Moreover, some included studies lacked information and data, especially with respect to cfDNA integrity index: ALU247/ALU115. Third, only full-text studies published in English and Chinese were included in this meta-analysis. Because the authors could not easily understand other languages. Therefore, a potential selection bias may exist.

## MATERIALS AND METHODS

### Search strategy

A prospective protocol was registered on PROSPERO International prospective register of systematic reviews (identification number CRD42016047066). According to the Preferred Reporting Items for Systematic Reviews and Meta Analyses (PRISMA) [28], we conducted meta-analyses and reported the results.

We performed a systematic literature search in several electronic databases, including PubMed, EMBASE, Cochrane Library and Chinese National Knowledge Infrastructure (CNKI) from inception to August 07, 2017

The search terms were as follows: (“colorectal neoplasms/diagnosis”[Mesh OR ((cancer OR neoplasm OR tumor OR carcinoma) AND (colon OR rectal OR colorectal))) AND (cell free DNA OR circulating DNA OR cfDNA) AND (blood OR serum OR plasma OR circulation) AND (diagnoses OR sensitivity and specificity OR ROC curve).

In order to assess completeness, we also reviewed the reference lists from all included articles to identify additional relevant studies. No attempt was made to recover unpublished studies.

### Study selection

Eligible studies had to meet the following inclusion criteria:

(1) the outcome of interest was quantitatively analysis to the diagnostic accuracy of circulating cfDNA for colorectal cancer; (2) sensitivity and specificity were reported or could be calculated; (3) absolute numbers of true-positive (TP), false-positive (FP), true-negative (TN), and false-negative (FN) were provided;

Two reviewers (X Wang and XQ Shi) independently determined the eligibility of the studies, and disagreements in decisions were resolved by consensus.

### Data extraction

The following data were extracted from each identified study by two reviewers (X Wang and XQ Shi): last name of the first author; study location; publication year; number of cases and controls; methods of detection; type of specimens; measuring objects; cut off values; diagnostic performance, including sensitivity, specificity, TP, FP, TN, and FN.

### Quality assessment

We used Quality Assessment of Diagnostic Accuracy Studies-2 (QUADAS-2) [29] to assess the methodological quality of each study and potential risk of bias by two reviewers (X Wang and PW Zeng).

### Statistical analysis

Referring to the standard methods of previous diagnostic meta-analysis [24], we calculated the pooled sensitivity, specificity, diagnostic odds ratio (DOR), positive likelihood ratio (PLR), and negative likelihood ratio (NLR) by the bivariate model. Simultaneously, the summarized receiver operating characteristic (SROC) curve were generated by plotting the sensitivity and specificity of each of the included studies [30]. The area under the curve (AUC) was used for judging the diagnostic value and accuracy as a potential summary of the SROC curve [31]. In addition, the threshold effect was examined to assesse the heterogeneity among studies by the Spearman's correlation coefficient, a value of p less than 0.05 indicated significant threshold effect and heterogeneity, and there was a negative correlation between sensitivity and specificity.

The Higgins I^2^ statistics were also used to assess the heterogeneity between studies. A random-effects model was applied when significant heterogeneity was detected. We considered a value of p less than 0.1 or an I^2^ value > 50% to indicate substantial heterogeneity [32]. Subgroup analysis and meta-regression analyses were performed to explore the potential sources of between-study heterogeneity. Moreover, we created Deeks’ funnel plots asymmetry test to detect publication bias (*p* < 0.10) [33]. Clinical utility of the cfDNA was evaluated by the Fagan nomogram.

All statistical analyses were performed by Meta-DiSc 1.4 (Cochrane Colloquium, Barcelona, Spain) and STATA 12·0 statistical software package (Stata Corporation, College Station, Texas, USA). A threshold of *p* < 0·05 was considered statistically significant except where otherwise specified.

## CONCLUSIONS

Our meta-analysis suggested that the diagnostic accuracy of circulating cfDNA has unsatisfactory sensitivity but acceptable specificity for diagnosis of colorectal cancer. Furthermore, the integrity index (ALU247/ALU115) is better than absolute DNA concentration in diagnostic accuracy of colorectal cancer. We hold the opinion that the cfDNA may be valuable in early complementary diagnosis, but still need to be combined with conventional examination for colorectal cancer detection. Additional highquality rigorous studies, especially, comparing multiple time points in different tumor stage group and employing reasonable measurement techniques, should be performed to confirm our results or explore the clinical utility of cfDNA in the diagnosis of colorectal cancer.

## SUPPLEMENTARY MATERIALS FIGURES AND TABLES


