# Supplementary material for: Circulating cell free DNA as the diagnostic marker for colorectal cancer: a systematic review and meta-analysis
Source: Oncotarget. 2018 May 11;9(36):24514–24. doi: 10.18632/oncotarget.25314 (PMC5966252; doi:10.18632/oncotarget.25314)
Supplement: Supplementary file 1 [file oncotarget-09-24514-s001.pdf]

Circulating cell free DNA as the diagnostic marker for colorectal cancer: a systematic review and meta-analysis

SUPPLEMENTARY MATERIALS

List of abbreviations

CEA: Carcinoembryonic Antigen; CA 19-9: Carbohydrate Antigen-19-9; CfDNA: Cell Free DNA; PROSPERO: prospective registration of systematic reviews; PRISMA: Preferred Reporting Items for Systematic Reviews and Meta Analyses; EMBASE: Excerpta Medica Database; CNKI: Chinese National Knowledge Infrastructure; TP: True-Positive; FP: False-Positive; TN: True-Negative; FN: False-Negative; QUADAS-2: Quality Assessment of Diagnostic Accuracy Studies-2; DOR: Diagnostic Odds Ratio; PLR: Positive Likelihood Ratio; NLR: Negative Likelihood Ratio; SROC: Summarized Receiver Operating Characteristic; AUC: Area Under the Curve; RT(q)PCR: Real-Time(quantitative) polymerase chain reaction; RFS: Recurrences-Free Survival; OS: Overall Survival; HR (95% CI): Hazard Ratio (95% Confidence Interval).

Supplementary Table 1: I^2 and *p* values for individual subgroup analysis

Variables	No. of data		Sensitivity	Specificity	PLR	NLR	DOR
**Overall**	18	I_2	88.6	82.8	80.8	89.1	73.8
		P	0.000	0.000	0.000	0.000	0.000
**Measuring object**							
**ALU247/ALU115**	3	I_2	84.3	65.4	66.5	80.7	68.6
		P	0.002	0.056	0.051	0.006	0.041
**ALU115&CFDlevels**	13	I_2	89.5	85.0	83.6	90.1	75.7
		P	0.000	0.000	0.000	0.000	0.000
**Country**							
**China**	6	I_2	92.3	13.0	21.7	93.1	48.1
		P	0.000	0.332	0.271	0.000	0.086
**Italy**	5	I_2	63.4	79.6	68.3	60.0	64.3
		P	0.027	0.001	0.013	0.040	0.025
**Other countries**	7	I_2	85.7	58.6	52.9	76.9	55.7
		P	0.000	0.025	0.048	0.000	0.035
**Sample**							
**Plasma**	5	I_2	94.7	70.7	44.1	94.5	61.2
		P	0.000	0.008	0.128	0.000	0.036
**serum**	13	I_2	83.1	85.6	85.9	86.1	78.2
		P	0.000	0.000	0.000	0.000	0.000
**sample size**							
**≥ 100**	7	I_2	92.2	76.9	73.5	93.0	67.3
		P	0.000	0.000	0.001	0.000	0.005
**< 100**	11	I_2	86.2	85.1	77.9	84.5	73.1
		P	0.000	0.000	0.000	0.000	0.000
